# Comparison of diets for Largemouth and Smallmouth Bass in Eastern Lake Ontario using DNA barcoding and stable isotope analysis

**DOI:** 10.1371/journal.pone.0181914

**Published:** 2017-08-03

**Authors:** Erich J. H. Nelson, Jeremy Holden, Robert Eves, Bruce Tufts

**Affiliations:** 1 Freshwater Fisheries Conservation Lab, Department of Biology, Queen’s University, Kingston, Ontario, Canada; 2 Lake Ontario Management Unit, Glenora Fisheries Station, Ontario Ministry of Natural Resources and Forestry, Picton, Ontario, Canada; 3 Protein Function Discovery Lab, Department of Biomedical and Molecular Sciences, Queen’s University, Kingston, Ontario, Canada; University of Guelph, CANADA

## Abstract

Largemouth (LMB: *Micropterus salmoides*) and Smallmouth Bass (SMB: *Micropterus dolomieu*) are important species in the recreational fisheries of the Laurentian Great Lakes. The invasion of the Round Goby (*Neogobius melanostomus*) into these lakes has changed several facets of black bass biology, but there is still much to learn about the relationship between these species. Previous dietary analyses have shown Round Goby to be important prey for bass, but have been limited by low visual identification rates of dissected stomach items. Within the present study, DNA barcoding and stable isotope analysis improve prey identification and provide a more quantitative dietary analysis of adult black bass in Lake Ontario, comparing the importance of Round Goby as prey between these two species. Eighty-four LMB (406mm fork length ±4mm SEM) and two hundred sixty-four SMB (422mm ±2mm) obtained as tournament mortalities had prey identified using DNA-based methods. Round Goby was the most prevalent prey species for both predators. The diet of LMB was three times more diverse than that of SMB, which almost entirely consists of Round Goby. Our results provide further support that recent increases in the size of Lake Ontario bass are a result of Round Goby consumption, and that the effects of this dietary shift on body condition are greater for SMB. Techniques developed in this study include reverse-oriented dual priming oligonucleotides used as blocking primers for predator DNA, and an automated design approach of restriction fragment length polymorphism tests for identifying prey DNA barcodes.

## Introduction

North America’s recreational fisheries provide substantial economic benefits for many local communities [1]. In the Great Lakes region, the economic impact of recreational fisheries is approximately $8.3 billion per annum [2]. Black bass are among the most popular species for North American freshwater anglers and the most common target species of a growing element of recreational fisheries, competitive fishing events [3,4]. Evidence indicates that the number of competitive fishing events (tournaments) for bass in the Great Lakes has been increasing in recent years [5].

Invasive species have had a profound influence on the ecology of the Great Lakes and St. Lawrence River ecosystems [6]. One prolific invader, the Round Goby (*Neogobius melanostomus*) [7], has affected many aspects of black bass ecology including: diet [8], body condition [9], nesting success [10] and population size [11]. Most previous studies in this area have focused on the interactions between Round Goby and Smallmouth Bass (SMB: *Micropterus dolomieu*). There are also important recreational fisheries for Largemouth Bass (LMB: *Micropterus salmoides*) in areas where Round Goby are now common, such as the Eastern basin of Lake Ontario and the St. Lawrence River, but there is currently much less known about the interactions between these two species.

Although previous studies have shown that many Great Lakes piscivores are now including Round Goby in their diet, quantitative data of diets for different life stages is necessary for a thorough understanding of the impact of this invasive species on food webs. To date, a thorough quantitative assessment of the diet of large adult bass, using modern DNA-based approaches, is lacking for populations in the Great Lakes. Furthermore, studies in this area have relied on a visual approach for taxonomic identification, which has several limitations compared to DNA-based identification. For example, traditional visual approaches to diet analysis leave many prey items unidentified and can overlook rare species [12]. Modern DNA-based approaches greatly increase the success rate of identification and provide more accurate estimations of dietary contribution [13,14]. Due to sampling limitations, previous studies in both this area and ongoing assessment programs also contain relatively few large adult bass.

The extent of digestion varies greatly between prey items extracted from fish stomachs [15], with a large proportion being too degraded for physical identification [16]. Semi-digested soft tissue usually has intact mitochondrial DNA for PCR amplification of species-specific barcodes [17]. Aligning these barcode sequences with known voucher specimens can be used to increase diet resolution upward of 90% [18,19]. This approach relies on some *a priori* knowledge of prey, and the use of taxon-specific primers, to amplify a 652bp segment from the 5’ region of *cytochrome c oxidase I* (COI) [20], which can then be identified with minimal error using a molecular database [21]. For adult black bass, most prey fall into two categories, freshwater fish or crayfish, with each taxa having successfully tested primer cocktails available for DNA barcoding [22,23]. Using a restriction fragment length polymorphism (RFLP) method to identify prey [24,25] instead of sequencing by capillary electrophoresis of PCR products [26], will make it possible to achieve species level resolution of most prey more efficiently. RFLP tests are useful in diet studies aiming to identify a single clade of prey, and since Round Goby is expected to be the dominant prey species for LMB and SMB, using an RFLP test will allow for faster identification. For this study, we will also sequence PCR products that were shown by RFLP analysis to not be Round Goby to achieve a more complete analysis of bass diets.

The use of barcode analysis to determine the diet of piscivorous fish has the potential to amplify predator DNA alongside prey due to their genetic similarity [27]. It is therefore possible that cannibalism could go undetected with this approach [28]. Co-amplification of predator and prey DNA can also result in ambiguous sequence reads. Predator-specific restriction enzymes or 3’ carbon spacers can be used to increase purity of prey PCR products when these cases occur [29,30]. Blocking oligonucleotides can also improve the purity of prey DNA, for both RFLP and direct sequencing approaches to diet analysis, by selectively limiting PCR amplification of predator DNA [31]. To the best of our knowledge, DNA barcoding has only been applied once to identify prey of LMB in South Korea [32], and blocking primers have not been developed for either species of bass.

Stable carbon (δ^13^C) and nitrogen (δ^15^N) isotope ratios in animals undergo uniform enrichment between consumption events [33,34]. Analysis of these variables provides another modern approach for diet analysis, allowing for an assessment of primary producers [35] and a measure of trophic position [36]. Combining stable isotope data with a DNA-based approach can further resolve the energy flow of an ecosystem [37], and provide greater insight of changes in food web dynamics [38].

The main objective of this study is to assess the impact of the invasive Round Goby on the trophic ecology of large adult black bass in the Eastern Lake Ontario and the St. Lawrence River using DNA barcoding and stable isotope analysis. In this study we evaluate the impact of Round Goby by comparing (1) their relative importance in the diets of LMB and SMB, and (2) the body condition of bass before and after the Round Goby’s introduction. We hypothesize that Round Goby comprise a greater proportion of SMB diets because of the similar habitat preferences between these 2 species [39,40], which has led to a larger increase in the body condition of SMB compared to LMB. Secondary objectives of this study are to determine restriction enzymes for the rapid identification of Round Goby prey, and design blocking primers to limit PCR co-amplification of bass COI. We predict that DNA barcoding should greatly increase identification rates of prey and provide superior information to visual taxonomy. This study takes advantage of competitive fishing events in the region in order to obtain samples from tournament mortalities. This approach is not only cost effective and efficient, but also allows unique biological information to be acquired from the largest size class of bass in these fisheries. These fish make the greatest contribution to the next generation of their populations [41], but are difficult to sample in large numbers using other approaches.

## Materials and methods

### Sample collection

Incidental mortalities of 84 LMB and 264 SMB were collected from licenced fishing tournaments between 2012 and 2015, and stored at -20°C until further processing. Licenced tournaments were held by the following organizations: Canadian Bass Anglers Federation, Competitive Sport Fishing League, Quinte Fishing Series, and Renegade Bass Tour. Bass were angled from across the Eastern Basin of Lake Ontario and St. Lawrence River for tournaments in the following Canadian cities: Trenton (44°05'55.1"N 77°34'18.0"W), Belleville (44°09'19.0"N 77°22'52.2"W), Deseronto (44°11'29.7"N 77°03'09.5"W), Kingston (44°13'46.0"N 76°28'38.8"W), Gananoque (44°19'17.9"N 76°09'50.8"W), Rockport (44°22'57.2"N 75°55'42.0"W), and Morrisburg (44°53'34.7"N 75°10'45.9"W). Permits for collecting bass for scientific purposes were obtained from the Ontario Ministry of Natural Resources and Forestry. Before processing, bass were thawed at room temperature and measured for length and weight. Stomach contents were then dissected, separated into unique prey items, weighed, photographed, identified, and had soft tissue samples removed for DNA extraction. Skeletal muscle samples from each bass were also taken for subsequent analysis of stable isotope ratios. Stomach contents and skeletal muscle samples were returned to -20°C after dissection.

### Visual identification

Variation in digestion state of each prey item was categorized according to a five-tier system for visually assessing diet diversity in piscivorous fish [17], expanded to apply to crayfish remains. These categories are as follows; (1) empty stomach; (2) digested prey, no intact identifying characteristics; (3) digested prey, identifiable above species level; (4) digested prey, species identifiable; and (5) fully intact prey. Prey ranked between (5) and (3) were identified to the lowest taxonomic unit (LTU) using field guides [42,43] and trained personnel. Prey classified between ranks (2) and (4) were identified to the LTU using vertebrae for fish and chitinous body segments for crayfish. Using fresh razorblades and holding trays, ≤25mg soft tissue samples for DNA-based identification were taken from all prey items (LMB: n = 49; SMB: n = 287). All visually identified prey underwent our DNA barcoding approach to verify its accuracy and compare identification success rates.

### DNA barcode amplification

Genomic DNA was extracted from frozen tissue samples with the DNeasy Blood and Tissue Kit (Qiagen) according to the manufacturer’s tissue protocol. When required, quality and concentration of DNA extractions were determined with 2% agarose TBE electrophoresis gels run at 200 V for 0.3 h in a Sub-Cell GT Cell (Bio-Rad Laboratories) or a Nanodrop 1000 spectrophotometer (Thermo Fisher Scientific). PCRs and restriction digests took place within a T100 Thermal Cycler (Bio-Rad Laboratories).

PCR amplification occurred in 12.5 μl reactions consisting of; 2 μl DNA template; 10% D-(+)-trehalose; 0.25 mM dNTPs; 0.1 μM forward primers; 0.1 μM reverse primers; 1x DreamTaq Green Buffer; and 0.3125 U DreamTaq DNA Polymerase (Thermo Fisher Scientific) [23]. The COI-3 primer cocktail (primers VF2_t1, FishF2_t1, FishR2_t1 and FR1d_t1) was used for prey visually classified as fish, whereas the orcoCOI primer cocktail (primers orcoCOIF and orcoCOIR) was used for prey visually classified as crayfish (Table 1). The 16S primer cocktail (primers 16Sar-5’ and 16Sbr-3’) served as a positive control for DNA extraction for both fish and crayfish prey, as described by Ivanova et al. [23]. The Folmer primer cocktail (primers LCO1490 and HCO2198) was used in the event of poor COI primer specificity for both fish and crayfish prey (successful 16S amplification, and unsuccessful COI-3 or orcoCOI amplification).

**Table 1:** PCR primers for COI and 16S rDNA.

Name	Sequence	Reference
VF2_t1	TGTAAAACGACGGCCAGTCAACCAACCACAAAGACATTGGCAC	[44]
FishF2_t1	TGTAAAACGACGGCCAGTCGACTAATCATAAAGATATCGGCAC	[44]
FishR2_t1	CAGGAAACAGCTATGACACTTCAGGGTGACCGAAGAATCAGAA	[44]
FR1d_t1	CAGGAAACAGCTATGACACCTCAGGGTGTCCGAARAAYCARAA	[23]
orcoCOIF	GTGGTAGTTACAGCYCATGC	[45]
orcoCOIR	CCAGACTCTTGAACTACAAT	[45]
16Sar-5’	CGCCTGTTTATCAAAAACAT	[46]
16Sbr-3’	CCGGTCTGAACTCAGATCACGT	[46]
LCO1490	GGTCAACAAATCATAAAGATATTGG	[47]
HCO2198	TAAACTTCAGGGTGACCAAAAAAT	[47]

Reaction conditions for the COI-3 and 16S cocktails were: 1 min at 94°C, 35 cycles of 30 s at 94°C, 40 s at 52°C and 60 s at 72°C, followed by 10 min at 72°C [23]. Conditions for the orcoCOI cocktail were: 2 min at 95°C, 40 cycles of 30 s at 95°C, 30 s at 52°C and 60 s at 72°C, followed by 10 min at 72°C [45]. Conditions for the Folmer cocktail were: 1 min at 95°C, 5 cycles of 40 s at 94°C, 40 s at 45°C and 60 s at 72°C, 35 cycles of 40 s at 94°C, 40 s at 51°C and 60 s at 72°C, followed by 5 min at 72°C [48]. Success of all PCRs was evaluated by agarose gel electrophoresis, as described above, prior to DNA sequencing. In order to limit processing time and effort, successful COI amplicons of fish prey were first screened for Round Goby (the suspected dominant prey species) using an in-lab RFLP test.

### Round Goby RFLP identification

COI barcode vouchers (n = 2708) from the 112 fish species endemic to the sampling region [43] were obtained from Barcode of Life Data Systems (BOLD) [49] and analyzed in R using a script written for this study (see Data Accessibility). Recognition site frequency per species was computed using a list of 263 commercially available restriction enzymes, and searched for candidates based on relative recognition frequencies. A single enzyme, *SspI*, was found to cleave all Round Goby COI barcodes and no other species’, yielding 242 and 496bp fragments with the COI-3 primer cocktail, or 228 and 481bp fragments with the Folmer primer cocktail. Restriction digest of fish prey PCRs occurred in 12.5 μl reactions consisting of; 5 μl PCR product; 1x CutSmart Buffer; and 1 U SspI-HF (New England Biolabs), incubated for 30 min at 37°C. PCR and restriction digest success was evaluated by running total digest volumes in 2% agarose gels.

### DNA barcode sequencing

To verify that Round Goby populations did not have a variable allele on the RFLP cut site, 16 RFLP-positive Round Goby prey from this study underwent DNA sequencing to complement the 24 Round Goby barcode vouchers used to design the RFLP test [50]. In addition to the Round Goby prey, all crayfish prey (n = 10) and RFLP-negative fish prey (n = 45) also had PCR products purified with the GeneJET PCR Purification Kit (Thermo Fisher Scientific), and forward sequenced by capillary electrophoresis on an ABI 3730XL DNA Analyser (Applied Biosystems) in an external facility (TCAG, Canada). Tailed amplicons produced with the COI-3 cocktail were sequenced with M13 forward primers (Invitrogen); all other PCR products were sequenced with forward primers used in their amplification reaction. Sequence data was trimmed and exported from electropherograms using FinchTV (from Geospiza; available at geospiza.com/finchtv) and identified with BOLD Systems’ nearest neighbour algorithm using a 1% divergence threshold [49]. In the case of ambiguous results involving few closely related species, those not endemic to the study region were ruled out as possibilities. BOLD identifications were corroborated by the top results of a BLASTn search for highly similar sequences on GenBank [51].

### Predator DNA removal

Prey items classified as fish that were identified as the same species as the predator following DNA sequencing (n = 40) were considered for an alternative predator DNA removal approach before resequencing. No suitable enzymes for RFLP tests were identified for either species of black bass; however, *BglII* and *NsiI* in parallel digests were identified to both cut COI of only LMB and SMB. Due to the added complexity of eliminating predator DNA by this restriction digest approach, it was not favoured over alternative solutions. Three predator-specific annealing blocking primers were designed for LMB to improve the relative amplification of rare prey DNA (Table 2). These primers were selected to overlap the 3’ binding sites of reverse primers for LMB COI, limiting PCR amplification of predator DNA with its C3 spacer and 10x concentration relative to other reverse primers [29]. Dual priming oligonucleotides (DPO) were used to allow for longer oligonucleotides with unchanged melting temperatures, increasing the potential length of binding site overlap and allowing for greater target specificity. SMB was not specifically considered for primer design as a result of its larger sample size, low observed prey diversity and genetic similarity to LMB.

**Table 2:** COI blocking primers for LMB (*M*. *salmoides*) (I = deoxyinosine, 3 = C3 spacer).

Name	Sequence	Melting Temperature
COIR-blkMsa	ACCAGAATAAGTGCTGGTAAAGA3	55.6°C
COIR-DPO-blkMsa	GGTGGCCAAAGAACCAGAATIIIIICTGGTAAAGA3	55.6°C
COIR-DPOr-blkMsa	GGTGGCCAAAIIIIIAGAATAAGTGCTGGTAAAGA3	48.4°C

Using DNA extracted from bass skeletal muscle and voucher specimens of Round Goby and Alewife (*Alosa pseudoharengus*) (Glenora Fisheries Station, Canada), each of the blocking primers were tested on 100-fold excess mixtures of predator and prey DNA in COI-3 and Folmer PCRs. For each PCR amplicon, 5 μl was digested with *NsiI*¸ which has one COI recognition site in both LMB and SMB, producing 179 and 559bp fragments, but no COI recognition sites in Round Goby or Alewife. Restriction digest conditions were identical to the Round Goby RFLP protocol, using NsiI-HF (New England Biolabs) in lieu of Ssp-HF. Blocking efficiency was estimated by agarose gel electrophoresis of total digest volumes. DNA extractions that yielded amplicons sequence-identified as LMB or SMB underwent secondary COI-3 or Folmer PCRs with 1.0 μM of the most effective blocking primer added to the reaction mix.

### Stable isotope analysis

Skeletal muscle samples of approximately 50 mg were taken for stable isotope analysis from 15 LMB and 42 SMB subsampled from the set of tournament mortalities. Samples were freeze-dried for 24h, ground to a powder, then analyzed for δ^13^C, δ^15^N, %C and %N using an Elemental Analyzer (Costech)—Isotope Ratio Mass Spectrometer (Thermo Delta V) at an external facility (GLIER, Canada). Standards of acetanilide, bovine, poplar leaf, and glycine with verified values for δ^13^C and δ^15^N were included after every 12^th^ sample in each run. Every 12^th^ sample was also performed three times to verify consistency of instrumentation, and C:N ratios used to ensure normalization of the lipid affect across samples [52,53]. Trophic position (TP) was calculated for each fish with the equation TP_consumer_ = [(δ^15^N_consumer_−δ^15^N_baseline_)/3.4] + 2, using an δ^15^N baseline value of 8.5 ‰ [36, 38].

### Condition factor

The present study also included data from bass collected by the Ontario Ministry of Natural Resources and Forestry (OMNRF) assessment programs. A standardized fish community assessment program, consisting of bottom set gillnetting and bottom trawl tows, has been routinely conducted by the OMNRF since 1992 at fixed sites throughout the Bay of Quinte and the Eastern Basin of Lake Ontario [54,55]. Trap netting has also been conducted within the Bay of Quinte since 2001. The trap net survey follows a standardized Nearshore Community Index Netting (NSCIN) protocol [56] and program specific details are described by Hoyle and Yuille [57]. The present study used data for length and weight of 910 SMB from the gillnetting program as well as 166 SMB and 408 LMB captured in the trap netting program for determining condition factor. This data was used to show how the size distribution of tournament-sampled bass differs from the size distribution of the bass captured in these programs and to show how the condition factor has changed in LMB and SMB since the invasion of Round Goby into Lake Ontario.

### Data analysis

First and second order error of the Round Goby RFLP test were estimated using visual and barcode species identifications to corroborate restriction digest results. Percent index of relative importance (%IRI) was calculated for each species of prey identified in tournament-sampled bass stomachs, selected as the best measure of dietary importance factors as shown by Liao et al. [58]. %IRI was calculated as a percent of total importance to provide a comparable statistic between predator species [59,60], based on the formula *IRI*_*i*_ = %*O*_*i*_ * (%*W*_*i*_ + %*N*_*i*_). %O represents the percent occurrence of the prey species out of all bass stomachs, %W the percent of total weight comprised by the prey species (uncorrected for digestion), and %N the percent number of the prey species out of the total number of all prey. To contrast the alpha diversity of prey between bass species, true diversity profiles showing effective species number were calculated based on numerical prey composition [61–63]. The alpha diversity indices of q = 1 and q = 2 were specifically stated as they correspond to the more widely known Shannon’s and Simpson’s indices respectively. Relative weight was estimated using total length for tournament-sampled LMB (*W* = 10^−5.528^ * *L*^3.273^) and SMB (*W* = 10^−5.329^ * *L*^3.200^) [64,65]. Fulton’s condition factor was used to determine body condition for OMNRF-sampled bass using fork length [66], and analyzed using ANOVA for differences between species, collection period (pre- and post-Round Goby introduction) and sampling method.

## Results

### Sample collection

**Fig 1:**
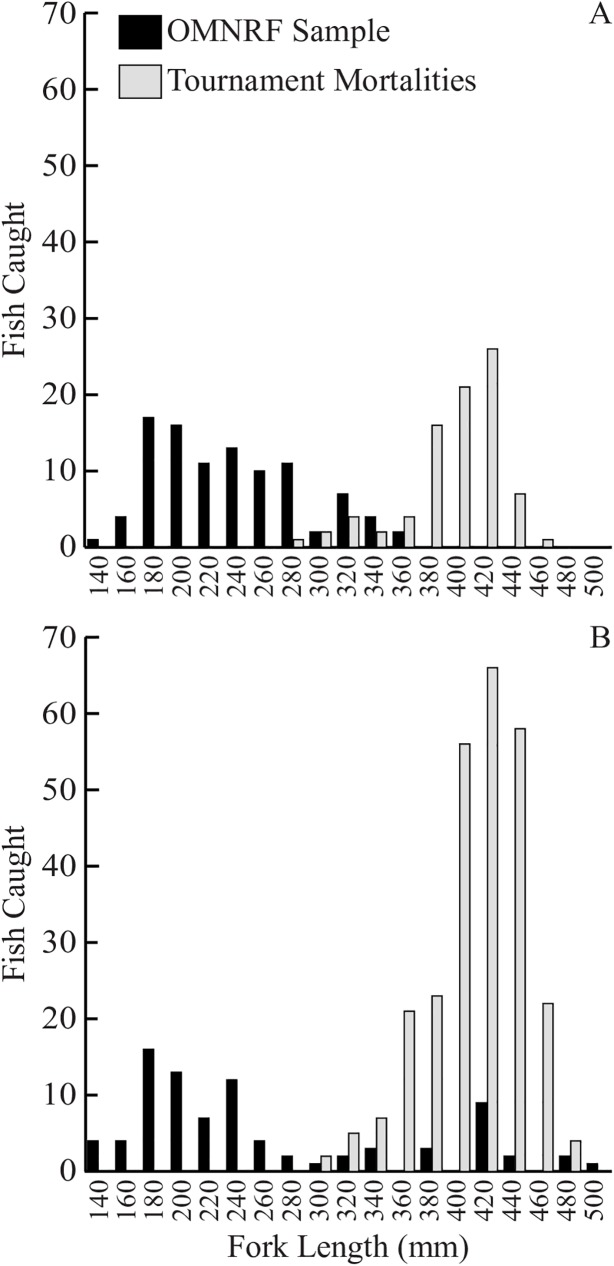
Comparison of fork length distributions for bass by collection method. Fork length distributions of (A) LMB and (B) SMB obtained from tournament mortalities (black: n = 348) or OMNRF gillnet and trap net samples (grey: n = 183) over a three-year period.

The data show that tournament mortalities provide a much greater number of large adult bass for both species when compared to more traditional sampling approaches (gillnet, trap net) over the same timeframe (Fig 1). During the three years that tournament mortalities were collected for each species, there were 55 LMB and 207 SMB that were greater than 400 mm in length. Over the same three-year timeframe, the standard assessment approaches used by OMNRF did not catch any LMB that were greater than 400mm and only caught 14 SMB above this length. The mean lengths of tournament mortalities (LMB: 406 mm ±4mm for LMB; SMB: 422 mm ±2mm) were significantly greater than the mean lengths of bass caught by OMNRF using gillnets and trapnets (LMB: 246 mm ±5mm; SMB: 268 mm ±11mm) (Welch two sample t-test, SMB: t = -14.326, df = 90.782, P<0.001; LMB: t = -24.493, df = 168.52, P<0.001).

### Visual identification vs DNA barcoding

**Fig 2:**
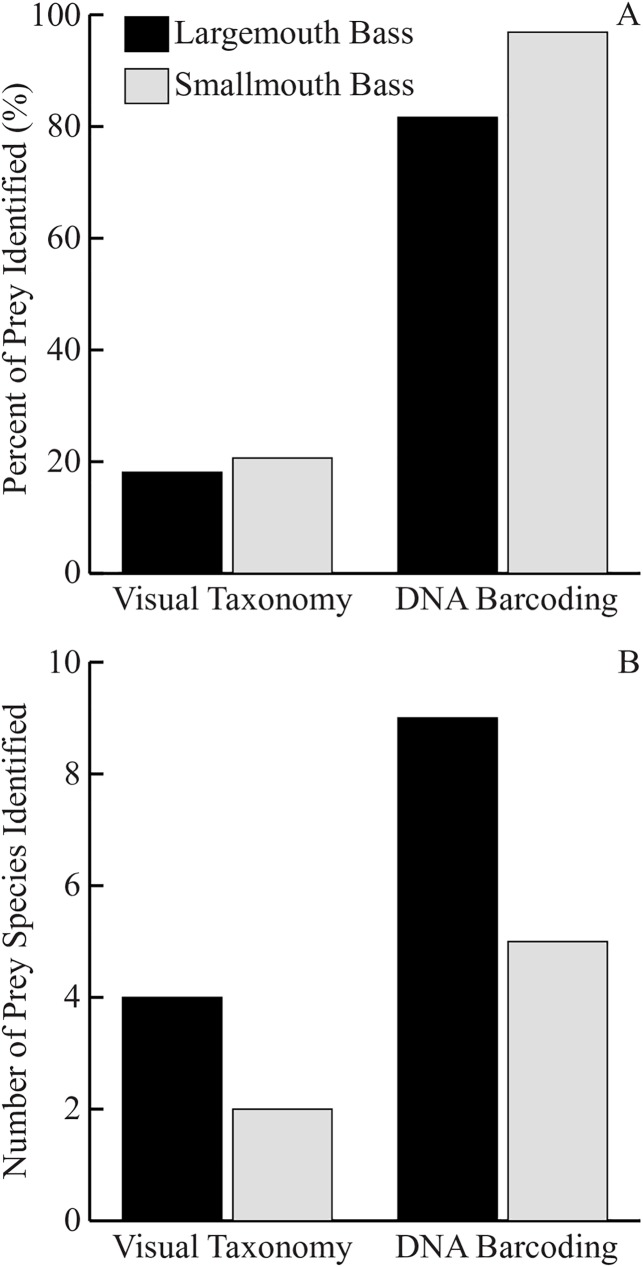
Comparison of bass stomach items identified using visual taxonomy or DNA barcoding. Comparison of percent stomach items identified (A) and number of species identified (B) in LMB (n = 84) and SMB (n = 264) using either visual taxonomy or DNA barcoding.

The results of this study showed the effectiveness of DNA barcoding as a tool to identify prey in the stomach contents of black bass. Of the 68 prey items visually identified to species in this study, all 68 returned the same species following identification by DNA barcoding (Table 3). The DNA approach also greatly increased species identification within bass stomach contents compared to visual identification (Fig 2, Table 3). Using visual identification, only 10% and 22% of prey could be identified in LMB and SMB respectively. In contrast, the percent of prey identified using DNA barcoding was 82% in LMB and 97% in SMB. Furthermore, DNA barcoding identified several species that were not found using the visual identification approach, including 5 new prey species for LMB, and 3 new prey species for SMB.

**Table 3:** Prey classification and identification success according to the ranking system; (1) empty stomach; (2) digested prey, no intact identifying characteristics; (3) digested prey, identifiable above species level; (4) digested prey, species identifiable; and (5) fully intact prey. Amplification of COI or 16S mitochondrial DNA was used as a positive control for DNA extraction.

Class	Number Classified		Visually Identified		DNA Extracted		Barcode Identified	
	LMB	SMB	LMB	SMB	LMB	SMB	LMB	SMB
1	46 (55%)	97 (37%)	-	-	-	-	-	-
2	35	175	0	0	27	167	27	167
3	9	49	0	0	9	49	8	48
4	5	63	5	63	5	63	5	63
5	0	0	0	0	0	0	0	0

### Round Goby RFLP identification

The Round Goby RFLP test designed in this study greatly reduced overall processing costs and time of DNA barcoding, as 268 of 336 total prey were identified to be Round Goby. Visual identification and DNA sequencing confirmed the species identity for 119 of the 336 total prey that underwent the RFLP test (Table 4). All prey classified as fish that failed the Round Goby RFLP test (n = 44) were identified as a different species upon barcode sequencing. Almost all (99%) successful DNA extractions could be identified using the BOLD Animal Identification System (http://boldsystems.org/index.php/IDS_OpenIdEngine), revealing 10 prey species across both species of bass. Of the 268 Round Goby RFLP identifications, 75 prey items were confirmed using visual identification (n = 61), DNA sequencing (n = 11) or a combination of both (n = 3). In designing the RFLP test, all Round Goby COI vouchers (n = 24) were found to have an *SspI* recognition site in the same location, whereas no COI vouchers (n = 2684) of the other 111 fish species contained an *SspI* recognition site.

**Table 4:** Abundance of prey within bass stomachs, indicated by occurrence out of all bass, total weight, and total number of prey items identified. Fish and crayfish data shown below includes all category (2) prey that failed DNA extraction but were visually identifiable as fish or crayfish.

Prey Species	Occurrence		Weight (g)		Number	
	LMB	SMB	LMB	SMB	LMB	SMB
*Alosa pseudoharengus*	5	21	41.9	79.9	6	24
*Ambloplites rupestris*	1	0	10.3	0	1	0
*Ameiurus nebulosus*	1	0	13.4	0	1	0
*Amia calva*	1	0	1.3	0	1	0
*Lepomis gibbosus*	2	1	24.1	7.8	3	1
*Lepomis macrochirus*	6	0	72.7	0	7	0
*Neogobius melanostomus*	15	144	81.6	1110.5	17	251
*Notemigonus crysoleucas*	1	0	0.3	0	1	0
*Orconectes propinquus*	2	1	7.2	1.9	3	1
*Orconectes virilis*	0	1	0	3.5	0	1
Fish	38	166	270.9	1203.3	44	282
Crayfish	3	4	14.8	16.8	5	5

### Predator DNA removal

**Fig 3:**
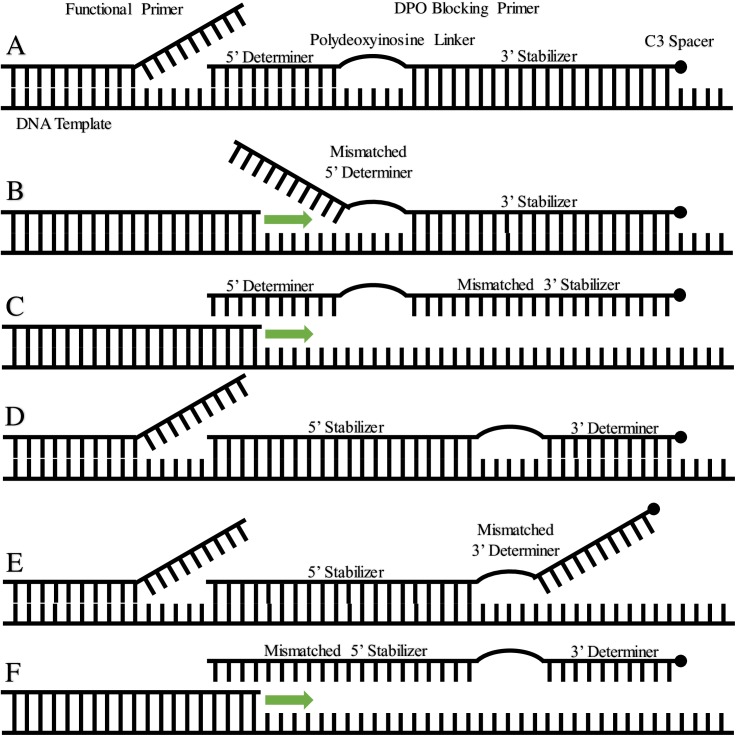
**Schematic outlining PCR elongation results of DPO blocking primer mismatch for reversed (A-C) and conventional (D-F) determiner and stabilizer orientation.** (A) Annealing to predator DNA, blocking elongation, as shown by COIR-DPOr-blkMsa with LMB in this study. (B) Partial annealing to non-predator DNA with mismatched 5’ determiner. Blocking primer functions as elongation arrest, not significantly reducing PCR amplification [29]. (C) No annealing to non-predator DNA due to mismatched stabilizer region [67]. (D) Annealing to predator DNA, blocking elongation, as shown by COIR-DPO-blkMsa with LMB in this study. (E) Partial annealing to non-predator DNA with mismatched 3’ determiner, blocking elongation, as shown by COIR-DPO-blkMsa with SMB in this study. (F) No annealing to non-predator DNA due to mismatched stabilizer region [67].

Blocking primer tests and primer alignment with mitochondrial genomes of LMB (Accession: NC_008106.1) and SMB (Accession: NC_011361.1) indicate only the 5’ stabilizer segment of COIR-DPO-blkMsa needs to bind to a DNA template to inhibit its PCR amplification with the COI-3 or Folmer primer cocktails. Mismatch of the shorter 3’ determiner segment performs the same function as the C3 spacer, resulting in no elongation when only the 5’ stabilizer has a correct binding site, as with SMB COI. Primer COIR-DPOr-blkMsa, with reversed stabilizer (3’) and determiner (5’) orientation to COIR-DPO-blkMsa, demonstrated limited amplification with only LMB COI. Previous work has shown elongation arrest blocking primers to have little success at blocking elongation [29], suggesting that ideal DPO blocking primers should have a 5’ determiner and 3’ stabilizer, and be positioned relative to functional primers such that binding site overlap occurs within the 3’ stabilizer (Fig 3). This design necessitates binding of the entire blocking primer for amplification to be inhibited, as opposed to just the 5’ stabilizer. Templates that bind only to the determiner are hypothesized to function as elongation arrests due to lack of functional primer overlap.

Blocking primer COIR-DPO-blkMsa was observed to inhibit COI amplification for LMB, SMB and Alewife, whereas COIR-DPOr-blkMsa and COIR-blkMsa only inhibited amplification for LMB. Since it was observed that the Folmer primer cocktail yielded no COI amplicon for LMB or SMB upon secondary sequencing for 40 prey items, this simpler process of amplifying prey COI in the presence of abundant predator DNA was favoured over the use of blocking primers.

### Diet composition

**Fig 4:**
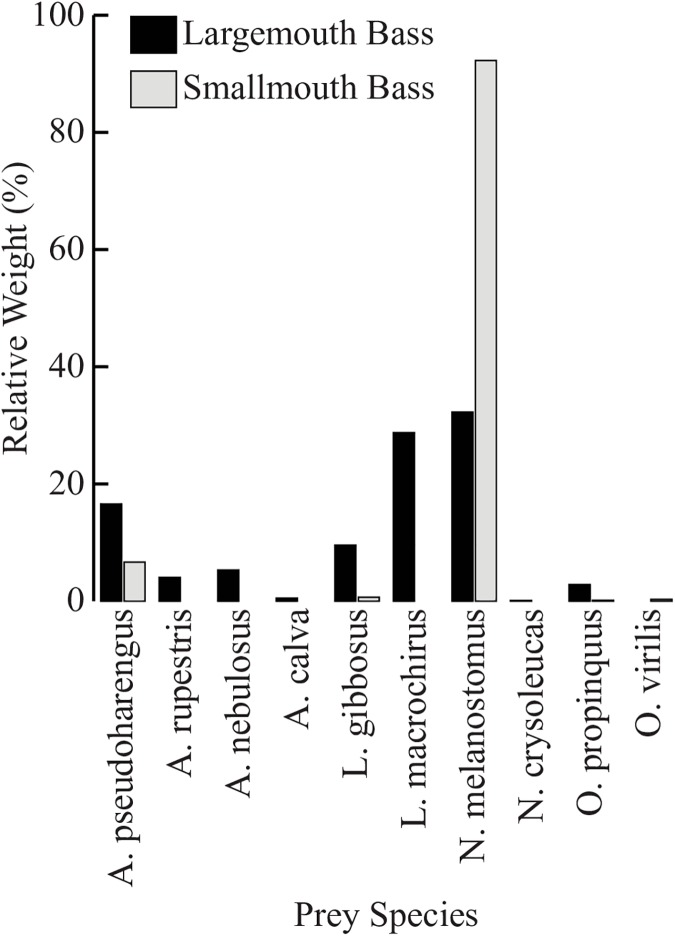
Percent wet weight of identified prey (LMB: n = 40; SMB: n = 278) from tournament-sampled LMB and SMB (LMB: n = 84; SMB: n = 264) for each prey species identified (n = 10).

**Fig 5:**
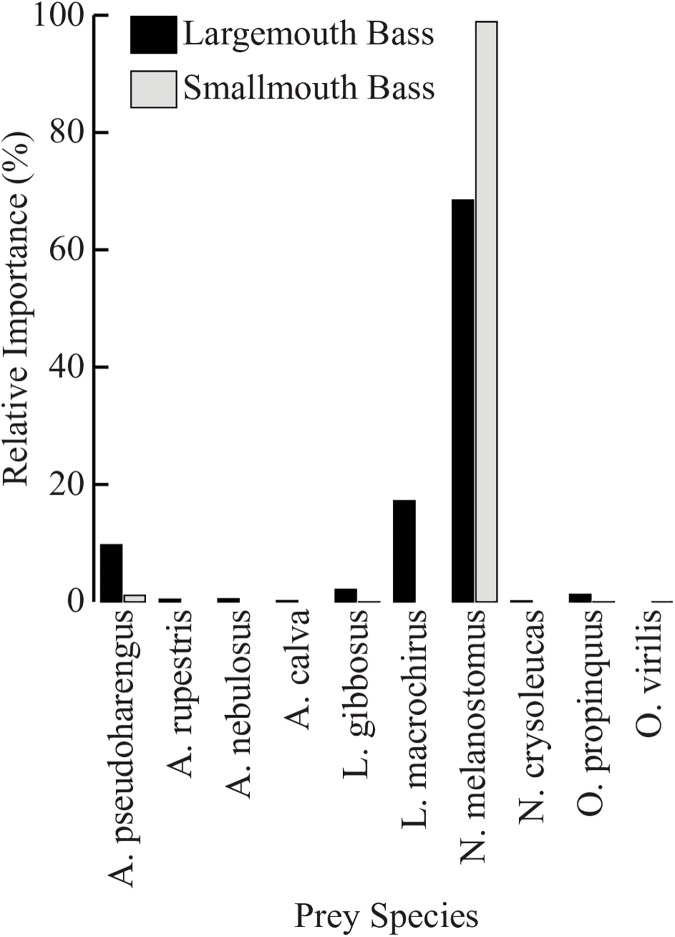
Percent index of relative importance of identified prey (LMB: n = 40; SMB: n = 278) from tournament-sampled LMB and SMB (LMB: n = 84; SMB: n = 264) for each prey species identified (n = 10).

**Fig 6:**
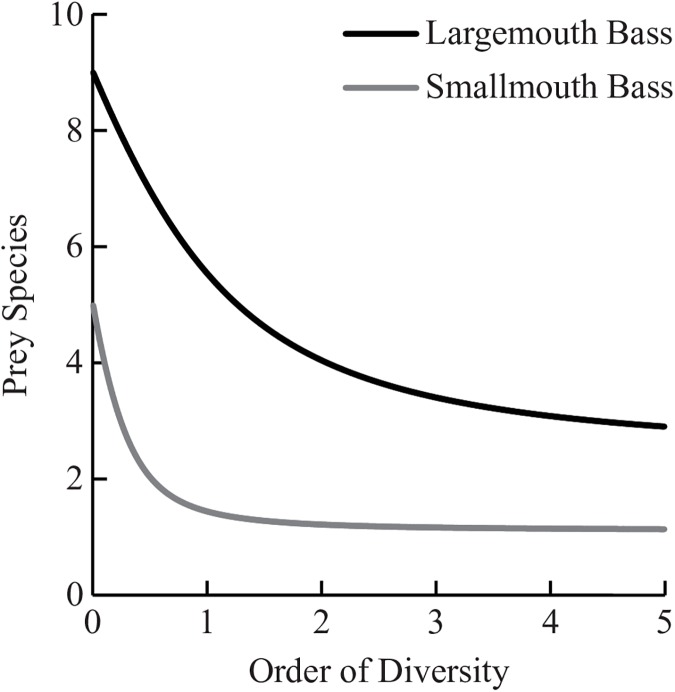
True diversity profiles (D) of identified prey (LMB: n = 40; SMB: n = 278) from tournament-sampled LMB and SMB (LMB: n = 84; SMB: n = 264) for each prey species identified (n = 10). Relative diversity is maximized at order q = 0.9, indicating LMB consume 3.8 times more prey species than SMB.

Diet analysis of tournament-sampled bass showed that there were significant differences between the two species. SMB had significantly more prey items per stomach (1.09 ±0.09 SE) than LMB (0.58 ±0.08) (t = -4.125, df = 270, P<0.001). No significant differences were found between average prey wet weight of LMB (5.83g ±0.83g) and SMB (4.25g ±0.22g) (t = 1.850, df = 55, P = 0.070), nor between total wet weight per stomach of SMB (4.62g ±0.36g) and LMB (3.40g ±0.67g) (t = -1.609, df = 135, P = 0.101). For both species of bass, Round Goby was the most abundant prey item by all measures (Table 4, Fig 4); however, Round Goby had a 31% greater index of relative importance for SMB diets than LMB diets (Fig 5). Over 95% of the relative importance of LMB prey can be attributed to 3 species: Round Goby, Bluegill Sunfish (*Lepomis machrochirus*), and Alewife. For both alpha diversity indices q = 1 (Shannon’s) and q = 2 (Simpson’s), LMB prey (^1^D = 5.53, ^2^D = 4.04) are over three times more diverse than SMB prey (^1^D = 1.47, ^2^D = 1.22), with the SMB prey community effectively consisting of one species (Fig 6).

### Stable isotope analysis

The trophic position of tournament-sampled LMB (4.10 ±0.06 SE) and SMB did not significantly differ from each other based on skeletal muscle tissue δ^15^N (t = 0.591, df = 16, P = 0.563). In terms of food chain bases, LMB were significantly less δ^13^C enriched at -24.37 ‰ ±0.76 (SE) than SMB at -19.88 ‰ ±0.20 (t = 5.682, df = 16, P<0.001).

### Condition factor

**Fig 7:**
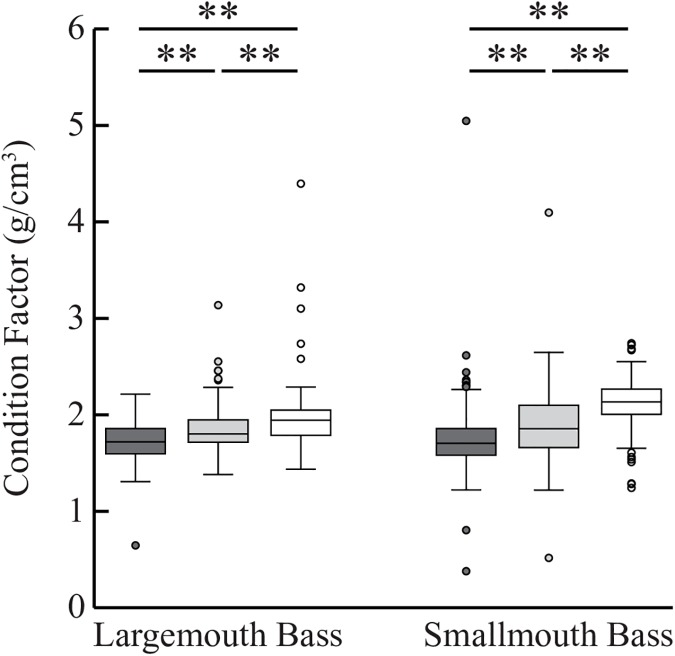
Bass condition factors before and after Round Goby invasion. Box plots showing condition factor for LMB and SMB, separated into pre-Round Goby (dark grey), post-Round Goby (light grey) OMNRF-sampled populations, and post-Round Goby (white) tournament-sampled populations (from left to right: n = 158, n = 253, n = 84, n = 757, n = 317, n = 264). Double asterisks (**) indicate p-values between 0.001 and 0.01 (Tukey’s post hoc test).

Within each species of bass, Fulton’s condition factor significantly differed between the pre-Goby and post-Goby OMNRF populations (ANOVA, LMB: F = 27.714, df = 494, P<0.001; SMB: F = 225.856, df = 1337, P<0.001) (Fig 7). Post-Goby populations of both species collected by the OMNRF had greater condition factor than pre-Goby populations (Tukey’s post hoc test, LMB: Q = 5.492, P<0.01; SMB: Q = 12.797, P<0.01). It is noteworthy that there was no significant difference between LMB and SMB condition factor for pre-Goby populations (t = 0.302, df = 270, P = 0.763). In post-goby populations, condition factor was significantly larger for SMB compared to LMB for both the OMNRF bass (t = -2.558, df = 546, P = 0.012) and the post-Goby tournament-sampled bass (t = -3.017, df = 102, P = 0.003). The post-Goby tournament-sampled bass also had higher condition factor than both pre-Goby and post-Goby bass populations sampled by the OMNRF (LMB: Q = (10.504, 6.842), P<0.01; SMB: Q = (29.674, 15.181), P<0.01).

## Discussion

Through the use of DNA barcoding for prey identification and sample collection from competitive fishing tournaments, this study was able to quantify and compare the diets of large adult LMB and SMB in Eastern Lake Ontario. Our results indicate that DNA barcoding has much higher resolution than traditional visual approaches for prey identification within stomach contents. Our results also show that collecting tournament mortalities was a very efficient approach for sampling the large adult bass in these populations that are not frequently captured using traditional assessment methods.

Using an RFLP test to identify Round Goby reduced processing time and costs within this study, while maintaining high identification fidelity. The approach left no room for false negative Round Goby identification, as all negative results were sequenced and identified using BOLD Systems, and gave no indication of false positives. In this study, 72% of identifications could be made in-lab using visual and RFLP analysis, leaving only 28% of identifications requiring DNA barcode sequencing at an external facility. Similar RFLP tests can be easily applied to other systems in which a large proportion of individuals belong to one species, and all potential prey species are known in advance. The R script used in this study can be used on barcode sequences obtained online to identify candidate restriction enzymes for the RFLP process.

Previous studies have shown that the invasive Round Goby has become an important food source for bass once it becomes established in waterbodies where bass are present. However, early attempts to quantify the importance of Round Goby in bass diets have been limited by low visual identification rates and small sample sizes of large adult bass. Using modern molecular approaches for diet analysis and tournament-sampled bass from Lake Ontario, our study was able to identify a greater number and a greater proportion of prey for adult bass than any previous study. Interestingly, this larger dataset shows a greater frequency of occurrence (86%) and relative importance (99%) of Round Goby in the diet of SMB in comparison to all previous studies that have included large adults since the Round Goby’s invasion of the Great Lakes [8,11,68–70]. Our results also indicate that the largest adults of the SMB population predominantly feed on Round Goby.

Since LMB are more tolerant of tournament practices than SMB [71], there are fewer tournament mortalities for LMB, decreasing our sample size relative to SMB. However, using the described molecular techniques we were able to obtain a high identification rate of prey items from these fish, and still gained valuable dietary information. Our results indicate that Round Goby have become an important prey item for LMB as well, but that this species maintains a more diverse diet than SMB. In the stomachs of LMB, we identified 9 different species, the most important of which were Round Goby, Bluegill Sunfish and Alewife. A previous study reported that Round Goby made up 88% by weight of the stomach contents of LMB in this region [8]. In comparison, the current study found that the percent composition of Round Goby in LMB stomachs was 29% by weight, with a 68% index of relative importance. The differences between our results and previous studies may be attributed to the fact that previous studies did not use DNA barcoding to identify prey (leading to lower taxonomic resolution and more unidentified prey items) and did not sample as many large adult bass.

The LMB and SMB collected at tournaments for this study had relative weights that were significantly greater (115.7% ±2.7% SE and 115.9% ±0.8% respectively) than the standard weights of these species elsewhere [64,65]. Crane and colleagues also recently reported that SMB from Lake Ontario had significantly greater mass-at-length following the Round Goby’s invasion [9]. Results from the present study confirm that there has been a change in the condition factor of SMB following the invasion of Round Goby into Lake Ontario. We also provide the first evidence that the condition factor in LMB has increased since the Round Goby’s invasion. Interestingly, the change in condition factor is somewhat less in LMB compared to SMB. This finding is consistent with our diet results showing that LMB still consume a variety of other species, and have a higher proportion of empty stomachs.

There are many differences in the ecology and behaviour of these two bass species, but the observed interspecific differences in diet may be largely explained by differences in their habitat preferences and feeding strategies [39,40]. Similar habitat preferences (rock and sand substrate) between SMB and Round Goby [72] may explain why this invasive species has had a greater influence on the trophic ecology of SMB. In contrast to SMB, LMB are a “sit and wait” predator with a preference for complex weedy habits, where there is likely more species diversity amongst potential prey.

Stable isotope ratios of carbon and nitrogen provide additional long-term information about diet and energy flow that is not obtainable from analyses of stomach contents. The trophic position of both LMB and SMB as determined by δ^15^N ratios did not differ from LMB (4.02 ±0.34 SD) and SMB (4.08 ±0.18) living in other freshwater Canadian lakes (n = 79, n = 21) not invaded by Round Goby [73]. On the other hand, lower δ^13^C enrichment in LMB indicated a more pelagic food chain basis in contrast to a more benthic basis for SMB, as would be predicted in lentic ecosystems such as Lake Ontario [74–76]. These differences suggest that Round Goby occupies the same trophic level as historically dominant prey items, such as Alewife in Lake Ontario [77], but forms a different benthic basis for energy flow in the food web. Similar research into the diets of piscivorous fish of the Baltic Sea following the invasion of Round Goby also indicate a consistent predatory fish trophic level, but a new primary source of carbon [78]. This change from pelagic to benthic feeding in SMB supports our hypothesis that LMB are not as dependent on Round Goby as SMB.

Using the Folmer primer cocktail to avoid co-amplification of bass with prey DNA negated the use of blocking primers in this study to obtain higher quality COI barcodes. As DNA could only be obtained from 41 of 49 LMB stomach items, and 10% of identified prey were the sole representatives of their species, future DNA analysis on dissected faeces has the potential to uncover greater diversity in the diet of this generalist predator. In addition to using the Folmer primer cocktail, blocking primer COIR-blkMsa in tandem with the COI-3 primer cocktail can be used to inexpensively amplify prey COI barcodes that the Folmer cocktail does not effectively bind to. For the purpose of LMB faecal analysis in Lake Ontario, barcode vouchers from BOLD Systems suggest non-DPO blocking primer COIR-blkMsa is sufficient to only inhibit amplification of LMB DNA.

In summary, this is the first quantitative evaluation, using a DNA-based approach, of the relative importance of the invasive Round Goby in the diets of large adult LMB and SMB from Eastern Lake Ontario. This molecular approach contributed to a larger sample size of identified prey than previous studies based on visual identification methods. Our results show that large adult SMB in Lake Ontario now feed almost exclusively on Round Goby during the summer months. Although adult LMB also consume large numbers of Round Goby, their diet remains more diverse than that of SMB. The shift to diets made up largely of Round Goby has led to an increase in body condition of both bass species, but this change is greater in SMB than LMB. According to stable isotope data, both bass species still reside at the same trophic level while maintaining ecologically distinct food chain bases. Future studies examining the impact of these changes in trophic ecology on important life history traits of these bass species, such as age at maturity, are warranted.

## References

[1] Tufts BL, Holden J, DeMille M. Benefits arising from sustainable use of North America’s fishery resources: economic and conservation impacts of recreational angling. International Journal of Environmental Studies. 2015;72(5):850–868.

[2] Fisheries and Oceans Canada. 2010 Survey of Recreational Fishing in Canada. Available from http://www.dfo-mpo.gc.ca/stats/rec/can/2010/RECFISH2010_ENG.pdf.

[3] Schramm HL Jr, Armstrong ML, Funicelli NA, Green DM, Lee DP, Manns RE Jr, et al The status of competitive sport fishing in North America. Fisheries. 1991 5 1;16(3):4–12.

[4] Funnell E. The Smallmouth Bass in Ontario. Biodiversity Brach Ontario Ministry of Natural Resources, Peterborough, Ontario; 2012. p. 61.

[5] Kerr S. A survey of 2008 competitive fishing events in Ontario. Fisheries Policy Section Biodiversity Branch Ontario Ministry of Natural Resources, Peterborough, Ontario; 2008. p. 8.

[6] Hecky RE, Smith RE, Barton DR, Guildford SJ, Taylor WD, Charlton MN, et al The nearshore phosphorus shunt: a consequence of ecosystem engineering by dreissenids in the Laurentian Great Lakes. Canadian Journal of Fisheries and Aquatic Sciences. 2004;61(7):1285–1293.

[7] Kornis MS, Mercado‐Silva N, Vander Zanden MJ. Twenty years of invasion: a review of round goby Neogobius melanostomus biology, spread and ecological implications. Journal of Fish Biology. 2012 2 1;80(2):235–85. doi: 10.1111/j.1095-8649.2011.03157.x 22268429 10.1111/j.1095-8649.2011.03157.x

[8] Taraborelli AC, Fox MG, Johnson TB, Schaner T. Round goby (*Neogobius melanostomus*) population structure, biomass, prey consumption and mortality from predation in the Bay of Quinte, Lake Ontario. Journal of Great Lakes Research. 2010 12;36(4):625–32.

[9] Crane DP, Farrell JM, Einhouse DW, Lantry JR, Markham JL. Trends in body condition of native piscivores following invasion of Lakes Erie and Ontario by the round goby. Freshwater Biology. 2015 1 1;60(1):111–24.

[10] Steinhart GB, Marschall EA, Stein RA. Round goby predation on smallmouth bass offspring in nests during simulated catch-and-release angling. Transactions of the American Fisheries Society. 2004 1 1;133(1):121–31.

[11] Lantry JR. Eastern Basin of Lake Ontario Warmwater Fisheries Assessment, 1976–2014. 2014 annual report, Bureau of Fisheries, Lake Ontario Unit and St. Lawrence River Unit to the Great Lakes Fishery Commission’s Lake Ontario Committee; 2015. Sec. 4, pp. 1–35. Available from http://www.dec.ny.gov/docs/fish_marine_pdf/lorpt14.pdf.

[12] Moritz C, Cicero C. DNA barcoding: promise and pitfalls. PLOS Biology. 2004 9 28;2(10):e354 doi: 10.1371/journal.pbio.0020354 15486587 10.1371/journal.pbio.0020354 PMC519004

[13] Symondson WO. Molecular identification of prey in predator diets. Molecular Ecology. 2002 4 1;11(4):627–41. 11972753 10.1046/j.1365-294x.2002.01471.x

[14] Dunn MR, Szabo A, McVeagh MS, Smith PJ. The diet of deepwater sharks and the benefits of using DNA identification of prey. Deep Sea Research Part I: Oceanographic Research Papers. 2010 7 31;57(7):923–30.

[15] Molnár G, Tölg I. Relation between water temperature and gastric digestion of largemouth bass (*Micropterus salmoides Lacepede*). Journal of the Fisheries Board of Canada. 1962 6 1;19(6):1005–12.

[16] Jang MH, Joo GJ, Lucas MC. Diet of introduced largemouth bass in Korean rivers and potential interactions with native fishes. Ecology of Freshwater Fish. 2006 9 1;15(3):315–20.

[17] Carreon‐Martinez L, Johnson TB, Ludsin SA, Heath DD. Utilization of stomach content DNA to determine diet diversity in piscivorous fishes. Journal of Fish Biology. 2011 4 1;78(4):1170–82. doi: 10.1111/j.1095-8649.2011.02925.x 21463313 10.1111/j.1095-8649.2011.02925.x

[18] Barnett A, Redd KS, Frusher SD, Stevens JD, Semmens JM. Non-lethal method to obtain stomach samples from a large marine predator and the use of DNA analysis to improve dietary information. Journal of Experimental Marine Biology and Ecology. 2010 9 30;393(1):188–92.

[19] Méheust E, Alfonsi E, Le Ménec P, Hassani S, Jung JL. DNA barcoding for the identification of soft remains of prey in the stomach contents of grey seals (*Halichoerus grypus*) and harbour porpoises (*Phocoena phocoena*). Marine Biology Research. 2015 4 21;11(4):385–95.

[20] Hebert PD, Stoeckle MY, Zemlak TS, Francis CM. Identification of birds through DNA barcodes. PLOS Biology. 2004 9 28;2(10):e312 doi: 10.1371/journal.pbio.0020312 15455034 10.1371/journal.pbio.0020312 PMC518999

[21] Meyer CP, Paulay G. DNA barcoding: error rates based on comprehensive sampling. PLOS Biology. 2005 11 29;3(12):e422 doi: 10.1371/journal.pbio.0030422 16336051 10.1371/journal.pbio.0030422 PMC1287506

[22] Costa FO, DeWaard JR, Boutillier J, Ratnasingham S, Dooh RT, Hajibabaei M, et al Biological identifications through DNA barcodes: the case of the Crustacea. Canadian Journal of Fisheries and Aquatic Sciences. 2007 2 1;64(2):272–95.

[23] Ivanova NV, Zemlak TS, Hanner RH, Hebert PD. Universal primer cocktails for fish DNA barcoding. Molecular Ecology Notes. 2007 7 1;7(4):544–8.

[24] Kohout J, Pekárik L, Šedivá A, Didenko A, Čiampor F, Čiamporová‐Zaťovičová Z. Discrimination between invasive Ponto‐Caspian gobies using a PCR‐RFLP method. Journal of Applied Ichthyology. 2014 2 1;30(1):121–6.

[25] Paquin MM, Buckley TW, Hibpshman RE, Canino MF. DNA-based identification methods of prey fish from stomach contents of 12 species of eastern North Pacific groundfish. Deep Sea Research Part I: Oceanographic Research Papers. 2014 3 31;85:110–7.

[26] Armani A, Giusti A, Guardone L, Castigliego L, Gianfaldoni D, Guidi A. Universal Primers Used for Species Identification of Foodstuff of Animal Origin: Effects of Oligonucleotide Tails on PCR Amplification and Sequencing Performance. Food Analytical Methods. 2016 5 1;9(5):1199–209.

[27] Deagle BE, Tollit DJ, Jarman SN, Hindell MA, Trites AW, Gales NJ. Molecular scatology as a tool to study diet: analysis of prey DNA in scats from captive Steller sea lions. Molecular Ecology. 2005 5 1;14(6):1831–42. doi: 10.1111/j.1365-294X.2005.02531.x 15836654 10.1111/j.1365-294X.2005.02531.x

[28] Valdez-Moreno M, Quintal-Lizama C, Gómez-Lozano R, del Carmen García-Rivas M. Monitoring an alien invasion: DNA barcoding and the identification of lionfish and their prey on coral reefs of the Mexican Caribbean. PLOS ONE. 2012 6 1;7(6):e36636 doi: 10.1371/journal.pone.0036636 22675470 10.1371/journal.pone.0036636 PMC3365883

[29] Vestheim H, Jarman SN. Blocking primers to enhance PCR amplification of rare sequences in mixed samples–a case study on prey DNA in Antarctic krill stomachs. Frontiers in Zoology. 2008 7 20;5(1):1. 18638418 10.1186/1742-9994-5-12 PMC2517594

[30] Leray M, Agudelo N, Mills SC, Meyer CP. Effectiveness of annealing blocking primers versus restriction enzymes for characterization of generalist diets: unexpected prey revealed in the gut contents of two coral reef fish species. PLOS ONE. 2013 4 8;8(4):e58076 doi: 10.1371/journal.pone.0058076 23579925 10.1371/journal.pone.0058076 PMC3620324

[31] Murray DC, Bunce M, Cannell BL, Oliver R, Houston J, White NE, et al DNA-based faecal dietary analysis: a comparison of qPCR and high throughput sequencing approaches. PLOS ONE. 2011 10 6;6(10):e25776 doi: 10.1371/journal.pone.0025776 21998697 10.1371/journal.pone.0025776 PMC3188572

[32] Jo H, Gim JA, Jeong KS, Kim HS, Joo GJ. Application of DNA barcoding for identification of freshwater carnivorous fish diets: Is number of prey items dependent on size class for Micropterus salmoides?. Ecology and Evolution. 2014 1 1;4(2):219–29. doi: 10.1002/ece3.921 24558577 10.1002/ece3.921 PMC3925385

[33] DeNiro MJ, Epstein S. Influence of diet on the distribution of carbon isotopes in animals. Geochimica et cosmochimica acta. 1978 5 31;42(5):495–506.

[34] DeNiro MJ, Epstein S. Influence of diet on the distribution of nitrogen isotopes in animals. Geochimica et cosmochimica acta. 1981 3 31;45(3):341–51.

[35] Hecky RE, Hesslein RH. Contributions of benthic algae to lake food webs as revealed by stable isotope analysis. Journal of the North American Benthological Society. 1995 12 1:631–53.

[36] Vander Zanden MJ, Rasmussen JB. Food web perspectives on studies of bass populations in north-temperate lakes. In American Fisheries Society Symposium; 2002 Vol. 31, pp. 173–184.

[37] Carreon‐Martinez L, Heath DD. Revolution in food web analysis and trophic ecology: diet analysis by DNA and stable isotope analysis. Molecular Ecology. 2010 1 1;19(1):25–7. doi: 10.1111/j.1365-294X.2009.04412.x 20078768 10.1111/j.1365-294X.2009.04412.x

[38] Vander Zanden MJ, Shuter BJ, Lester N, Rasmussen JB. Patterns of food chain length in lakes: a stable isotope study. The American Naturalist. 1999 10;154(4):406–16. doi: 10.1086/303250 10523487 10.1086/303250

[39] Stuber RJ, Gebhart G, Maughan OE. Habitat suitability index models: largemouth bass. US Fish and Wildlife Service; 1982.

[40] Edwards EA, Gebhart G, Maughan OE. Habitat suitability information: smallmouth bass. Oklahoma Cooperative Fish and Wildlife Research Unit, Stillwater; 1983. Sep.

[41] Hixon MA, Johnson DW, Sogard SM. BOFFFFs: on the importance of conserving old-growth age structure in fishery populations. ICES Journal of Marine Science: Journal du Conseil. 2014;71(8):2171–2185.

[42] Crocker DW, Barr DW. Handbook of the crayfishes of Ontario. ROM; 1968.

[43] Holm E, Mandrak NE, Burridge ME. The ROM field guide to freshwater fishes of Ontario. ROM; 2009.

[44] Ward RD, Zemlak TS, Innes BH, Last PR, Hebert PD. DNA barcoding Australia's fish species. Philosophical Transactions of the Royal Society of London B: Biological Sciences. 2005 10 29;360(1462):1847–57. doi: 10.1098/rstb.2005.1716 16214743 10.1098/rstb.2005.1716 PMC1609232

[45] Mathews LM, Adams L, Anderson E, Basile M, Gottardi E, Buckholt MA. Genetic and morphological evidence for substantial hidden biodiversity in a freshwater crayfish species complex. Molecular Phylogenetics and Evolution. 2008 7 31;48(1):126–35. doi: 10.1016/j.ympev.2008.02.006 18346914 10.1016/j.ympev.2008.02.006

[46] Palumbi SR. Nucleic acids II: the polymerase chain reaction. Molecular systematics. 1996;2:205–47.

[47] Folmer O, Black M, Hoeh W, Lutz R, Vrijenhoek R. DNA primers for amplification of mitochondrial cytochrome c oxidase subunit I from diverse metazoan invertebrates. Molecular Marine Biology and Biotechnology. 1994;3(5):294–9. 7881515

[48] Vidergar N, Toplak N, Kuntner M. Streamlining DNA barcoding protocols: automated DNA Extraction and a new cox1 primer in arachnid systematics. PLOS ONE. 2014 11 21;9(11):e113030 doi: 10.1371/journal.pone.0113030 25415202 10.1371/journal.pone.0113030 PMC4240537

[49] Ratnasingham S, Hebert PD. BOLD: The Barcode of Life Data System (http://www.barcodinglife.org). Molecular Ecology Notes. 2007 5 1;7(3):355–64. doi: 10.1111/j.1471-8286.2007.01678.x 18784790 10.1111/j.1471-8286.2007.01678.x PMC1890991

[50] Hale ML, Burg TM, Steeves TE. Sampling for microsatellite-based population genetic studies: 25 to 30 individuals per population is enough to accurately estimate allele frequencies. PLOS ONE. 2012;7(9):e45170 doi: 10.1371/journal.pone.0045170 22984627 10.1371/journal.pone.0045170 PMC3440332

[51] Johnson M, Zaretskaya I, Raytselis Y, Merezhuk Y, McGinnis S, Madden TL. NCBI BLAST: a better web interface. Nucleic acids research. 2008 7 1;36(suppl 2):W5–9. 18440982 10.1093/nar/gkn201 PMC2447716

[52] Logan JM, Jardine TD, Miller TJ, Bunn SE, Cunjak RA, Lutcavage ME. Lipid corrections in carbon and nitrogen stable isotope analyses: comparison of chemical extraction and modelling methods. Journal of Animal Ecology. 2008 7 1;77(4):838–46. doi: 10.1111/j.1365-2656.2008.01394.x 18489570 10.1111/j.1365-2656.2008.01394.x

[53] Mintenbeck K, Brey T, Jacob U, Knust R, Struck U. How to account for the lipid effect on carbon stable‐isotope ratio (δ13C): sample treatment effects and model bias. Journal of Fish Biology. 2008 3 1;72(4):815–30.

[54] Hoyle JA, Bowlby JN, Brousseau CM, Johnson TB, Morrison BJ, Randall RG. Fish community structure in the Bay of Quinte, Lake Ontario: The influence of nutrient levels and invasive species. Aquatic Ecosystem Health & Management. 2012;15(4):370–384.

[55] Hoyle JA. Fish species composition, distribution and abundance trends in the open-coastal waters of northeastern Lake Ontario, 1992–2012. Aquatic Ecosystem Health & Management. 2015;18(1):89–100.

[56] Stirling MR. Manual of instructions: Nearshore fish community index netting (NSCIN). Sutton (ON): Ontario Ministry of Natural Resources. Science and Information Branch; 1999.

[57] Hoyle JA, Yuille MJ. Nearshore fish community assessment on Lake Ontario and the St. Lawrence River: A trap net-based index of biotic integrity. Journal of Great Lakes Research. 2016;42(3):687–694.

[58] Liao H, Pierce CL, Larscheid JG. Empirical assessment of indices of prey importance in the diets of predacious fish. Transactions of the American Fisheries Society. 2001 7 1;130(4):583–91.

[59] Cortés E. A critical review of methods of studying fish feeding based on analysis of stomach contents: application to elasmobranch fishes. Canadian Journal of Fisheries and Aquatic Sciences. 1997 3 1;54(3):726–38.

[60] Hart RK, Calver MC, Dickman CR. The index of relative importance: an alternative approach to reducing bias in descriptive studies of animal diets. Wildlife Research. 2003 1 20;29(5):415–21.

[61] Jost L. Entropy and diversity. Oikos. 2006 5 1;113(2):363–75.

[62] Tuomisto H. A consistent terminology for quantifying species diversity? Yes, it does exist. Oecologia. 2010 12 1;164(4):853–60. doi: 10.1007/s00442-010-1812-0 20978798 10.1007/s00442-010-1812-0

[63] Leinster T, Cobbold CA. Measuring diversity: the importance of species similarity. Ecology. 2012 3 1;93(3):477–89. 22624203 10.1890/10-2402.1

[64] Henson JC. Quantitative description and development of a species-specific standard growth form for largemouth bass with application to the relative weight (Wr) index. Ph. D. Dissertation, Texas A&M University. 1991.

[65] Kolander TD, Willis DW, Murphy BR. Proposed revision of the standard weight (Ws) equation for smallmouth bass. North American Journal of Fisheries Management. 1993 5 1;13(2):398–400.

[66] Nash RD, Valencia AH, Geffen AJ. The origin of Fulton’s condition factor—setting the record straight. Fisheries. 2006 5 1;31(5):236–8.

[67] Chun JY, Kim KJ, Hwang IT, Kim YJ, Lee DH, Lee IK, Kim JK. Dual priming oligonucleotide system for the multiplex detection of respiratory viruses and SNP genotyping of CYP2C19 gene. Nucleic acids research. 2007 3 1;35(6):e40 doi: 10.1093/nar/gkm051 17287288 10.1093/nar/gkm051 PMC1874606

[68] Steinhart GB, Stein RA, Marschall EA. High growth rate of young-of-the-year smallmouth bass in Lake Erie: a result of the round goby invasion?. Journal of Great Lakes Research. 2004 12 31;30(3):381–9.

[69] Johnson TB, Bunnell DB, Knight CT. A potential new energy pathway in central Lake Erie: the round goby connection. Journal of Great Lakes Research. 2005 12 31;31:238–51.

[70] Reyjol Y, Brodeur P, Mailhot Y, Mingelbier M, Dumont P. Do native predators feed on non-native prey? The case of round goby in a fluvial piscivorous fish assemblage. Journal of Great Lakes Research. 2010 12 31;36(4):618–24.

[71] Furimsky M, Cooke SJ, Suski CD, Wang Y, Tufts BL. Respiratory and circulatory responses to hypoxia in largemouth bass and smallmouth bass: implications for “live-release” angling tournaments. Transactions of the American Fisheries Society. 2003 11 1;132(6):1065–75.

[72] Ray WJ, Corkum LD. Habitat and site affinity of the round goby. Journal of Great Lakes Research. 2001 12 31;27(3):329–34.

[73] Vander Zanden MJ, Cabana G, Rasmussen JB. Comparing trophic position of freshwater fish calculated using stable nitrogen isotope ratios (δ15N) and literature dietary data. Canadian Journal of Fisheries and Aquatic Sciences. 1997 5 1;54(5):1142–58.

[74] Doi H, Kikuchi E, Shikano S, Takagi S. Differences in nitrogen and carbon stable isotopes between planktonic and benthic microalgae. Limnology. 2010 8 1;11(2):185–92.

[75] Nerot C, Lorrain A, Grall J, Gillikin DP, Munaron JM, Le Bris H, et al Stable isotope variations in benthic filter feeders across a large depth gradient on the continental shelf. Estuarine, Coastal and Shelf Science. 2012 1 1;96:228–35.

[76] Freedman JA, Lorson BD, Taylor RB, Carline RF, Stauffer JR Jr. River of the dammed: longitudinal changes in fish assemblages in response to dams. Hydrobiologia. 2014 4 1;727(1):19–33.

[77] Paterson G, Rush SA, Arts MT, Drouillard KG, Haffner GD, Johnson TB, et al Ecological tracers reveal resource convergence among prey fish species in a large lake ecosystem. Freshwater Biology. 2014 10 1;59(10):2150–61.

[78] Almqvist G, Strandmark AK, Appelberg M. Has the invasive round goby caused new links in Baltic food webs?. Environmental biology of fishes. 2010 9 1;89(1):79–93.

